# How Long to Continue Eyelid Hygiene to Treat Meibomian Gland Dysfunction

**DOI:** 10.3390/jcm11030529

**Published:** 2022-01-20

**Authors:** Hyunmin Ahn, Bo Yi Kim, Jinyoung Kim, Yong Woo Ji, Ikhyun Jun, Tae-im Kim, Hyung Keun Lee, Kyoung Yul Seo

**Affiliations:** 1 Department of Ophthalmology, Severance Hospital, Yonsei University College of Medicine, Seoul 03722, Korea; overhyun31@gmail.com (H.A.); bykimm@yuhs.ac (B.Y.K.); lusita30@yuhs.ac (Y.W.J.); hadesdual@yuhs.ac (I.J.); tikim@yuhs.ac (T.-i.K.); shadik@yuhs.ac (H.K.L.); 2 Department of Medicine, Armed Forces Daegu Hospital, Daegu 38427, Korea; jnote0504@gmail.com; 3 Department of Internal Medicine, Asan Medical Center, University of Ulsan College of Medicine, Seoul 05505, Korea

**Keywords:** compliance, eyelid hygiene, meibomian gland dysfunction

## Abstract

To determine the efficacy duration of eyelid hygiene for meibomian gland dysfunction (MGD) treatment, a total of 1015 participants with primary MGD, followed for at least 6 months, were enrolled. The participants were classified into the eyelid hygiene group and the control group. The participants who had stopped eyelid hygiene at any point in the observation period after the initial 2 months were classified into the withdrawal group. Analysis was conducted with a generalized linear mixed model. Treatment group, age, sex, ocular surface inflammation, anti-inflammatory treatments, and baseline MGD subtype were considered as fixed effects, and the individual factor was considered as a random effect. The MGD stage decreased significantly for the observational period in the eyelid hygiene group (*p* < 0.001). Approximately 40.1% of the participants continuously maintained eyelid hygiene throughout the observational period. The MGD stage in the eyelid hygiene group continued to decrease for 6 months and was maintained thereafter. After 4 months of stopping eyelid hygiene, the MGD stage in the withdrawal group was worse than in the eyelid hygiene group (*p* < 0.001) and similar to that in the control group (*p* = 0.762). Maintaining eyelid hygiene was significantly effective in MGD treatment. Efficacy increased with treatment for 6 months, and the efficacy duration was maintained for 4 months even after stopping eyelid hygiene. Therefore, we recommend that patients with MGD maintain eyelid hygiene, and compliance should be checked continuously.

## 1. Introduction

Meibomian gland dysfunction (MGD) is a chronic, diffuse abnormality of the meibomian glands that may or may not be accompanied by qualitative and quantitative changes in secretion [1]. Eyelid margin inflammation, microorganism overgrowth, or other functional conditions can induce meibum quality changes and decrease meibum secretion, with or without atrophy of the secretory acini [2,3,4]. This condition results in an alteration of the tear film and ocular surface symptoms, and MGD is one of the major causes of dry eye disease (DED) [5]. MGD manifests as the result of various causes such as age, underlying disease, environment, and other factors [6]. This makes it difficult to treat MGD without further deterioration.

Eyelid hygiene is considered the first-line treatment for MGD [7,8]. The role of eyelid hygiene is to effectively remove inflammatory debris and enhance the expression of meibum through massage and warming [9]. Previous studies have determined that eyelid hygiene has a significant effect on MGD by comparative analyses between the baseline and follow-up periods [10,11], or between treatments [10,12,13]. Considering that MGD tends to worsen with age and the pathophysiology of MGD is a vicious circle [6], it may be important to maintain treatment.

However, no study has yet focused on the efficacy duration of eyelid hygiene, or determined whether the efficacy of eyelid hygiene continues without any deterioration after stopping. Furthermore, the speed at which MGD worsens remains unknown. Thus, this study sought to analyze the period of maximum efficacy and the time of recurrence after the discontinuation of eyelid hygiene for meibomian gland dysfunction.

## 2. Methods

This study was approved by the Medical Research Ethics Committee, Armed Forces Medical Command, Republic of Korea (protocol number AFMC-202104-HR-023–01). We screened patients over 18 years old who presented with dry eye to the Armed Force Daegu Hospital between May 2019 and April 2021 for inclusion in this observational study. The screened patients finished their interviews, and those who did not have a known systemic condition affecting the ocular surface (such as Stevens–Johnson syndrome) were enrolled. All research procedures were conducted in accordance with the principles of the Declaration of Helsinki.

### 2.1. Participants and Study Design

A total of 1015 patients were enrolled. This study was conducted on primary MGD only (Supplement Scheme S1). Patients with a known ophthalmic history causing dry eye and secondary MGD were excluded, as were patients with a Schirmer I test of <5 mm. For the design of the long-term observational study, the following participants were excluded: (1) follow-up less than 6 months, (2) failure to be defined in the initial 2-month compliance, (3) changed groups more than once, or (4) taking other treatments except eyelid hygiene and anti-inflammatory medications.

The participants were divided into two groups, the eyelid hygiene group and the control group, based on the initial 2-month eyelid hygiene performance. The participants were observed at 2-month intervals. Participants classified in the eyelid hygiene group at baseline who changed to the control group were defined as the withdrawal group. Compliance to eyelid hygiene was evaluated through a treatment diary, and the participants who performed eyelid hygiene at least once a day on average in the 2-month evaluation were selected as the eyelid hygiene group.

### 2.2. Meibomian Gland Dysfunction and Dry Eye Assessments

MGD was diagnosed using three indicators (eyelid margin abnormality, meibomian gland expressibility (MGE), and meibum quality (MQ)). For eyelid margin abnormalities, four signs (eyelid margin vascularity, plugged meibomian gland orifices, eyelid margin irregularity, and mucocutaneous junction shift) were checked [14]. The MGE score was assessed on a scale of 0 to 3 in five glands in the lower eyelid [7]. The MQ score was assessed in each of the eight glands of the central third of the lower eyelid on a scale of 0 to 3 for each gland, total score ranging from 0 to 24 [7]. The MGD stage was classified into five stages (0 to 4) following the MGD staging method from *The International Workshop on Meibomian Gland Dysfunction* [7], and stage 0 was defined when the MGE score was 0 and the MQ score was less than 2. The MGD stage was set as the primary outcome for the efficacy of eyelid hygiene. MGD was classified into four subtypes based on the MGE and the MQ scores [15]; the high-secretory (MGE score = 0) and low-secretory subtypes were determined by whether or not the MGE score was 0 and the high-quality (MQ score = 0) and low-quality were determined by whether or not the MQ score was 0.

The ocular surface disease index (OSDI), fluorescein tear break up time (FTBUT), Schirmer I test without anesthesia, and fluorescein corneal staining scores (CSS) were also measured. The OSDI questionnaire was scored on a scale of 0 to 100 [16]. CSS was obtained using the ocular staining score method on a scale of 0 to 3 [17]. Ocular surface inflammation was determined by the Efron bulbar conjunctival hyperemia scale [18], meibomitis, and lid margin vascularity.

### 2.3. Therapeutic Interventions

All participants were educated on eyelid hygiene practices for 10 min face-to-face at every visit. Patients were recommended to perform eyelid hygiene once or twice a day by washing with eyelid scrub products after warm compression for 5–10 min [19,20]. The compliance of eyelid hygiene was checked with treatment diary. Following the treatment guidelines for MGD with the ocular surface inflammation [20], cyclosporine 0.05% twice a day for 2 months and fluorometholone 0.1% twice a day for the initial 2–4 weeks were recommended as the anti-inflammatory treatment with prophylactic ciprofloxacin 0.5% 3–4 times a day [21,22,23,24,25]. Patients with other treatments for MGD were excluded (n = 61) (Supplement Scheme S1).

### 2.4. Statistical Analysis

To determine the efficacy duration of eyelid hygiene, the maximum efficacy period and the maintenance period after discontinuation were analyzed according to the MGD stage. The maximum efficacy period was defined as the visit time at which the MGD stage no longer decreased compared to the previous visit in the eyelid hygiene group. The efficacy duration after discontinuation of eyelid hygiene was defined as the withdrawal period when the MGD stage of the withdrawal group differed from that of the eyelid hygiene group and did not differ from that of the control group.

Because reflecting the time flow is key to this statistical analysis, a generalized linear mixed model with ordinal regression was used to analyze the efficacy of eyelid hygiene based on the MGD stage. Treatment group, age, sex, ocular surface inflammation, the anti-inflammatory treatments, and the baseline MGD type were considered as fixed effects, and the individual factor was considered as random effect. The working correlation matrix was set as an unstructured covariance model. The threshold for statistical significance was set at a *p*-value < 0.05. Pairwise comparisons to the follow-up periods were corrected using Tukey’s HSD method with the assumption of equal variance between the units of the stratified analyses.

## 3. Results

A total of 407/1015 (40.1%) patients with primary MGD who were observed for more than 6 months were consistently classified in the eyelid hygiene group. There was no significant difference in age, sex, ocular surface inflammation, anti-inflammatory treatments, MGD type, MGD stage, OSDI score, FTBUT, Schirmer I test, or CSS between the treatment groups in the initial 2 months (Table 1). The MGD stage and the MGD type according to age are described in Supplement Scheme S2. 

### Efficacy of Eyelid Hygiene and Factors Affecting the MGD Stage

**Figure 1:**
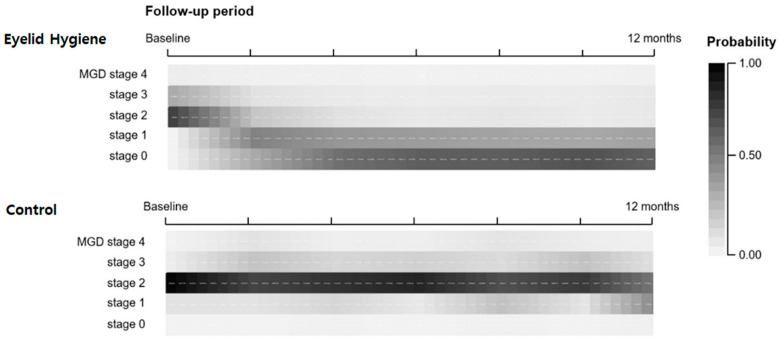
Heatmap charts showing the estimated probabilities of MGD stages in the treatment groups for 12 months.

With time consideration, the eyelid hygiene group showed significant decreases in the MGD stage (Table 2). During the 12-month follow-up, the interactions of the treatment groups and follow-up period were significantly different (ß = 0.764; 95% confidence interval (CI) 0.747–0.783; *p* < 0.001). Age and ocular surface inflammation increased the MGD stage (ß = 1.015; 95% CI 1.011–1.018; *p* < 0.001 and ß = 1.298; 95% CI 1.141–1.449; <0.001, respectively). The anti-inflammatory treatments and eyelid hygiene were negatively associated with the MGD stage (ß = 0.946; 95% CI 0.904–0.991; *p* = 0.001 and ß = 0.717; 95% CI 0.694–0.741; *p* < 0.001, respectively). Regarding the influence of the baseline MGD subtype, high-secretory high-quality MGD was the most effective compared to the other baseline MGD subtypes (*p* < 0.001). The estimated probability of the MGD stage by eyelid hygiene with treatment duration is demonstrated in Figure 1.

The impact of eyelid hygiene on the MGD stage in the eyelid hygiene group over the follow-up period is shown in Table 3. After consideration of the fixed effects, the MGD stage in the eyelid hygiene group continued to decrease until 6 months, not changing thereafter. With discontinuity of eyelid hygiene, the MGD stage of the patients in the withdrawal group was the same as that of the no eyelid hygiene group (*p* = 0.665), and there was a significant difference between the eyelid hygiene group and the withdrawal group in the MGD stage at 4 months (*p* < 0.001) (Table 4).

## 4. Discussion

One of the most frequent questions about eyelid hygiene from the patients with MGD in clinical practice is how long eyelid hygiene should be continued. This study could provide an answer to this question. In this study, the maximum efficacy of eyelid hygiene was continuously increased up to 6 months and maintained for 4 months after stopping eyelid hygiene. The efficacy duration of eyelid hygiene was considered with age, ocular surface inflammation, the anti-inflammatory treatments, and baseline MGD types. Thus, our study could help to explain how long eyelid hygiene should be performed in patients with MGD.

An unstable tear film caused by meibomian gland dysfunction can damage the ocular surface epithelium and lead to the expression of inflammatory cytokines. In turn, inflammatory disorders of the palpebral conjunctiva and lid margin may affect the structure and function of the meibomian gland [4]. As ocular surface inflammation is strongly associated with MGD in both cause and effect, anti-inflammatory treatments with steroids and/or cyclosporine could be considered [12,22]; only the time-limited anti-inflammatory treatment was permitted in this study. The MGD stage was improved over the course of the study, with the anti-inflammatory treatment demonstrating better results, although this difference was less than 10%, which was a similar result as the previous study [20,21].

Jiaxin et al. previously showed that the subtype of MGD classified by MGE and MQ could affect the clinical manifestation of DED [15]. Moreover, in this study, the MGD subtype affected the response to treatment. The turbidity of meibum and the expressibility of the meibomian gland could be considered. Further studies should be conducted to reveal the association between the MGD subtype and therapeutic response.

Only a few prior studies have investigated eyelid hygiene compliance. One study discussed that the efficacy of eyelid hygiene, which became less effective over time, was affected by patient compliance [26]. For this reason, to analyze the maximum efficacy or the duration of treatment efficacy, we suggest that therapeutic compliance must be well controlled in the study design. In this study, the compliance of eyelid hygiene was monitored with a treatment diary and statistical analyses were performed considering this compliance.

Only 40.1% of patients with MGD were compliant, even though face-to-face education regarding eyelid hygiene was provided at every visit in this study. One observational study reported that the compliance rate after 6 weeks was 55% [27]. However, the compliance included scrubbing using only water, relatively better accessibility than this study where the participants used eye scrub products. Unfortunately, the use of such products affects the course of eyelid hygiene, especially in the case of demodex-induced blepharitis [19]. One prior study quantitatively evaluated compliance by counting the procedures performed in a week, and the authors evaluated that the compliance was very good and thus did not reflect the compliance of eyelid hygiene [11]. The differences between the compliance rates in the studies might be due to the study design, and the fact that short-term experimental studies generally report a higher compliance rate than long-term observational studies [10,13,19]. In the actual clinical situation where long-term observation occurs, for this study, controlling the therapeutic compliance would provide more reliable results.

The study period was longer than that of previous studies, and patients with follow-up periods ranging from 6 months to 1 year were included. However, it is necessary to confirm the long-term effect of maintaining eyelid hygiene. In this respect, there was a limitation to this study, and further investigation is needed.

In accordance with the results of this study, we recommend performing eyelid hygiene for longer than 6 months depending on age, ocular surface inflammation, and MGD subtype. Even if the patient with MGD has stopped eyelid hygiene already and is doing well on their visit, careful follow-up for recurrence is required within 4 months.

## Figures and Tables

**Table 1:** Baseline characteristics of the study participants.

		Initial 2-Month Compliance Group of Eyelid Hygiene		
	Total (N = 1015)	Eyelid Hygiene (N = 543)	Control (N = 485)	*p*
Age (Mean ± SD)	38.7 ± 16.8	38.6 ± 16.5	38.8 ± 17.0	0.848
Sex (% of male)	72.1	75.6	74.2	0.462
Ocular surface inflammation (%)	69.2	67.4	71.2	0.196
Anti-inflammatory treatment (%)	58.0	57.3	58.8	0.629
***MGD parameters***				
MGD subtype (%)				0.069
High-secretory high-quality	39.9	39.1	40.9	-
High-secretory low-quality	33.0	34.6	31.1	-
Low-secretory high-quality	7.6	5.8	9.7	-
Low-secretory low-quality	19.5	20.5	18.3	-
MGD stage (%)				0.400
Stage 0	5.8	5.6	6.0	-
Stage 1	27.3	26.3	28.4	-
Stage 2	35.3	34.2	36.6	-
Stage 3	23.9	26.4	21.1	-
Stage 4	7.7	7.5	8.0	-
***DED parameters***				
OSDI scores (0–100)	17.7 ± 9.9	17.4 ± 10.1	18.0 ± 9.6	0.331
FTBUT (seconds)	7.4 ± 3.1	7.5 ± 3.2	7.2 ± 3.0	0.123
Schirmer I test (mm)	11.4 ± 4.3	11.4 ± 4.4	11.2 ± 4.0	0.448
Corneal staining score (%)				0.479
Score 0	21.9	23.2	20.4	-
Score 1	41.7	39.7	44.0	-
Score 2	21.5	22.4	20.5	-
Score 3	14.9	14.7	15.1	-

Abbreviations: DED, dry eye disease; FTBUT, fluorescein tear break up time; MGD, meibomian gland dysfunction; MQ, meibum quality; OSDI, ocular surface disease index.

**Table 2:** Influence of eyelid hygiene on the meibomian gland dysfunction stage.

		95% CI		
	Estimate β	Lower	Upper	*p*
***Fixed effects***				
Treatment group				
Eyelid Hygiene	0.717	0.694	0.741	<0.001 *
Control	Reference			
Follow-up (FU) period (each 2-month)	0.715	0.691	0.740	<0.001 *
Interaction between the groups and FU period				
Eyelid Hygiene	0.764	0.747	0.783	<0.001 *
Control	Reference			
Age	1.015	1.011	1.018	<0.001 *
Sex				
Male	0.968	0.862	1.082	0.560
Female	Reference			
Ocular surface Inflammation				
Present	1.298	1.141	1.449	<0.001 *
Absent	Reference			
Anti-inflammatory treatments				
Performed	0.946	0.904	0.991	0.001 *
Not performed	Reference			
MGD subtype, baseline				<0.001 *
Low-secretory low-quality	8.174	6.959	9.602	<0.001 *
Low-secretory high-quality	4.646	4.263	5.568	<0.001 *
High-secretory low-quality	2.852	2.316	3.532	<0.001 *
High-secretory high-quality	Reference			
***Threshold***				
MGD stage 4	55.257	43.948	77.478	<0.001 *
MGD stage 3	22.874	18.616	28.361	<0.001 *
MGD stage 2	9.885	8.240	11.882	<0.001 *
MGD stage 1	1.474	1.244	1.751	<0.001 *
MGD stage 0	Reference			

* Statistically significant.

**Table 3:** Impact of eyelid hygiene on meibomian gland dysfunction treatment duration.

	Treatment Duration					
Eyelid hygiene Group	2 months	4 months	6 months	8 months	10 months	12 months
	vs. baseline	vs. 2 months	vs. 4 months	vs. 6 months	vs. 8 months	vs. 10 months
Estimate ß	0.180	0.700	0.699	1.130	0.871	1.060
*p*-value	<0.001 *	0.002 *	0.004 *	0.345	0.312	0.610

* Statistically significant.

**Table 4:** Impact of discontinuity of eyelid hygiene on meibomian gland dysfunction by withdrawal period.

	Withdrawal Period					
Withdrawal Group	Baseline	2 months	4 months	6 months	8 months	10 months
vs. Control Group						
Estimate ß	0.255	0.460	0.890	0.904	1.065	1.081
*p*-value	<0.001 *	0.001 *	0.665	0.775	0.880	0.954
vs. Eyelid hygiene Group						
Estimate ß	0.900	1.656	5.112	5.284	5.212	5.171
*p*-value	0.712	0.122	<0.001 *	<0.001 *	<0.001 *	<0.001 *

* Statistically significant.

## Supplementary Materials

The following supporting information can be downloaded at: https://www.mdpi.com/article/10.3390/jcm11030529/s1, Scheme S1. Flow chart showing study participant selection. Scheme S2. The stages and types of meibomian gland dysfunction according to ages.
