# Supplementary material for: How Long to Continue Eyelid Hygiene to Treat Meibomian Gland Dysfunction
Source: J Clin Med. 2022 Jan 20;11(3):529. doi: 10.3390/jcm11030529 (PMC8837031; doi:10.3390/jcm11030529)
Supplement: Supplementary file 1 [file jcm-11-00529-s001.zip › jcm-1503894-supplementary.pdf]

Supplementary Figure Legends

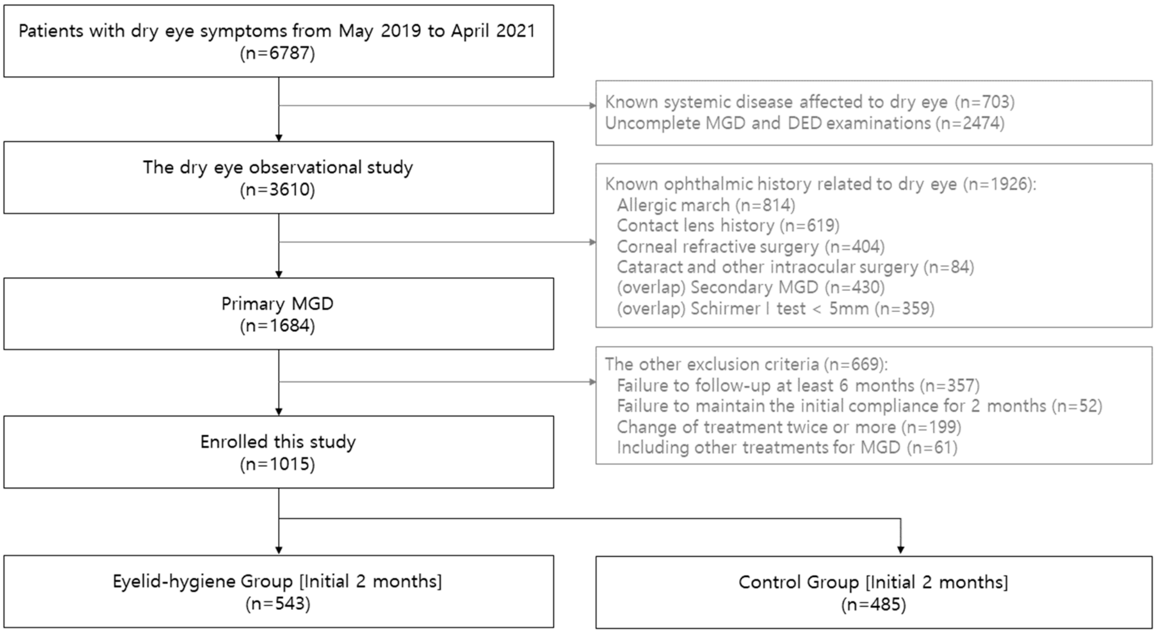

Scheme S1. Flow chart showing study participant selection.

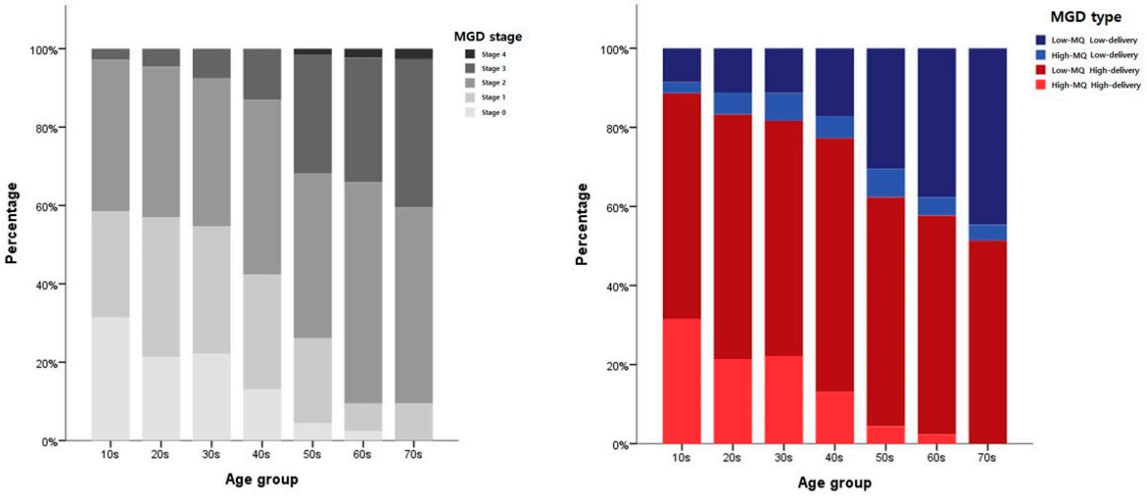

Scheme S2. The stages and types of meibomian gland dysfunction according to ages.
